# HIV-infected sex workers with beneficial HLA-variants are potential hubs for selection of HIV-1 recombinants that may affect disease progression

**DOI:** 10.1038/srep11253

**Published:** 2015-06-17

**Authors:** Chih-Hao Chang, Nicolaas C. Kist, Tammy L. Stuart Chester, Vattipally B. Sreenu, Melissa Herman, Ma Luo, Daniel Lunn, John Bell, Francis A. Plummer, T. Blake Ball, Aris Katzourakis, Astrid K. N. Iversen

**Affiliations:** 1 Medical Research Council Human Immunology Unit, Weatherall Institute of Molecular Medicine, University of Oxford, Oxford, United Kingdom; 2 Department of Zoology, University of Oxford, South Parks Road, Oxford, United Kingdom; 3 National HIV and Retrovirology Laboratories, JC Wilt Infectious Disease Research Centre, Winnipeg, Manitoba, Canada; 4 Department of Medical Microbiology, University of Manitoba, Winnipeg, MB, Canada; 5 Department of Statistics, University of Oxford, Oxford, United Kingdom; 6 Office of the Regius Professor of Medicine, The Richard Doll Building, University of Oxford, Oxford, United Kingdom; 7 Department of Immunology, University of Manitoba, Winnipeg, MB, Canada; 8 Nuffield Department of Clinical Neurosciences, Division of Clinical Neurology, Weatherall Institute of Molecular Medicine, University of Oxford, Oxford, United Kingdom

## Abstract

Cytotoxic T lymphocyte (CTL) responses against the HIV Gag protein are associated with lowering viremia; however, immune control is undermined by viral escape mutations. The rapid viral mutation rate is a key factor, but recombination may also contribute. We hypothesized that CTL responses drive the outgrowth of unique intra-patient HIV-recombinants (URFs) and examined gag sequences from a Kenyan sex worker cohort. We determined whether patients with HLA variants associated with effective CTL responses (beneficial HLA variants) were more likely to carry URFs and, if so, examined whether they progressed more rapidly than patients with beneficial HLA-variants who did not carry URFs. Women with beneficial HLA-variants (12/52) were more likely to carry URFs than those without beneficial HLA variants (3/61) (p < 0.0055; odds ratio = 5.7). Beneficial HLA variants were primarily found in slow/standard progressors in the URF group, whereas they predominated in long-term non-progressors/survivors in the remaining cohort (p = 0.0377). The URFs may sometimes spread and become circulating recombinant forms (CRFs) of HIV and local CRF fragments were over-represented in the URF sequences (p < 0.0001). Collectively, our results suggest that CTL-responses associated with beneficial HLA variants likely drive the outgrowth of URFs that might reduce the positive effect of these CTL responses on disease progression.

Millions of patients are already receiving expensive, life-long anti-HIV treatment, and millions more will require treatment because of the continued spread of HIV-1, particularly in resource-poor settings worldwide (www.unaids.org). The solution to this epidemic will require the development of a preventive vaccine and, potentially, therapeutic vaccines^1^. However, producing a safe and effective HIV-1 vaccine remains an elusive goal, and elucidating basic virus-host interactions is critical to improving vaccine strategies.

The immune system never successfully clears HIV-1 during the course of natural infection; however, in a few patients, the virus can be contained for decades through cytotoxic T lymphocyte (CTL) responses^2^. The protective CTL responses in these patients, who are known as long-term non-progressors (LTNPs), are primarily restricted by a few relatively rare human leukocyte antigen (HLA) variants, although not all patients with these HLA variants become LTNPs^2^,^3^,^4^,^5^. In addition, CTL responses against the HIV-1 capsid protein p24Gag are associated with lower mean viral loads in patients regardless of HLA restriction^6^. Because lower viral loads are associated with slower disease progression and lower transmission risk, these CTL responses improve the individual quality of life and limit the spread of the HIV-1 epidemic. However, the characteristics of an efficient CTL response and the reason why disease progression is not delayed in all patients with protective HLA variants remains unclear.

All CTL responses can select for mutations within or flanking CD8 epitopes, which allows HIV-1 to escape CTL recognition and prevents infected cells from being killed. These mutations may affect HLA binding, T cell receptor contact sites and/or antigen processing^2^,^7^,^8^,^9^,^10^,^11^. This evasion of immune responses is so advantageous that positive selection of escape mutations drives HIV-1 evolution within hosts^12^.

Escape mutations may occur relatively slowly and one-by-one in individual viruses over time or they may spread rapidly en-bloc through the HIV-1 population by recombination. Recombination occurs when the viral reverse transcriptase (RT) protein switches between the two HIV-1 RNA genomes and generates a recombinant DNA strand during transcription. *In vitro* studies have demonstrated that at least 2.8 crossovers occur per replication cycle per genome^13^; thus, the HIV-1 recombination rate per replication likely exceeds the RT mutation rate (3.4 × 10^−5^ mutations per nucleotide per cycle)^14^,^15^,^16^. However, because most infected cells carry only one integrated provirus^17^ the vast majority of co-packaged RNA genomes share a single provirus as a common ancestor. Therefore most recombination events occur between nearly identical sequences and are difficult to detect. Recombinants between more distantly related viruses are often not as fit as the parent viruses and selection in natural infections likely primarily favours the outgrowth of such recombinant viruses if recombination results in fitness gain^18^,^19^.

Most phylogenetic analyses of HIV-1 do not accommodate recombination because it violates the assumption of phylogeny inference of descent from a common ancestor^16^,^20^. However, recombination has been observed in acute HIV infection through visual inspection and/or comparisons with the inferred founder virus sequence^19^,^21^. This observation has been possible because one or a small number of viruses typically causes initial infection^19^,^21^,^22^,^23^. The risk of superinfection, i.e., a later infection by a second HIV strain, is similar to that of initial infection during the first 6 months and subsequently decreases by approximately half^24^,^25^ but can be difficult to recognize if the superinfecting HIV strain belongs to the same subtype as the HIV strain that initially infected the patient.

By contrast, recombination between two different HIV subtypes is easy to identify and the effect of recombination between subtypes is evident on the epidemiological scale. Most circulating HIV-1 strains belong to the main (M) group, which is divided into 10 homogeneous subtypes (labelled alphabetically), two sub-subtypes (e.g., HIV-A1 and HIV-A2) and several circulating recombinant forms (CRFs) of two or more subtypes (n > 60). Although the number of CRFs far exceeds the number of pure subtypes/sub-subtypes, the majority of HIV-infected people worldwide remain infected by a pure HIV subtypes/sub-subtypes, although CRFs may predominate in specific regions (e.g., CRF02_AG in West and West Central Africa^26^).

Because several HIV-1 strains co-circulate at high frequencies in some countries, e.g., Kenya^27^,^28^,^29^, high-risk patients in these regions are frequently infected by more than one subtype/sub-subtype or CRF. If a cell is superinfected by HIV-1 virions from different subtypes/sub-subtypes/CRFs^30^, two viral RNA genomes from different subtypes/sub-subtypes/CRFs can be packaged into the same virion in that cell (Fig. 1a). After this virion infects the next target cell, RT can generate an inter-subtype unique recombinant form (URF) of the HIV-1 subtypes/sub-subtypes/CRFs that can be readily identified (Fig. 1b)^31^.

Here, we hypothesized that selection favours the outgrowth of inter-subtype URFs in untreated, HIV-infected individuals if the URF contains advantageous changes that enable escape from CTL responses (Fig. 1c). Thus, we proposed that URFs would be more likely to be selected in hosts with CTL responses that are more efficient than average and that these recombinants would be likely to increase disease progression. Understanding the interplay between effective CTL responses and HIV-1 evolution is critical because these responses are the only anti-HIV immune responses known to be associated with delayed disease progression in natural infection^6^; thus, these responses are likely to be important to emulate in an HIV-1 vaccine. Furthermore, URFs have the potential to become CRFs and may have a critical effect on vaccine efficacy by increasing the global diversity of HIV-1.

To test our hypothesis, we examined the relationship between the HIV-1 form (homogeneous HIV-1 subtype/sub-subtype (HHS) or CRF versus URF), HLA profile and clinical progression rate in HIV-infected female commercial sex workers (CSWs) from the Pumwani slum in Nairobi, Kenya, where several HIV-1 subtypes and CRFs co-circulate^27^,^28^,^29^.

## Results

### HIV subtype distribution and recombination analyses

Approximately 1200 bases spanning the HIV-1 p17-p24 region from peripheral blood mononuclear cell (PBMC) DNA samples from each of the 113 patients from Pumwani were PCR amplified and sequenced using population sequencing to optimize primers followed by single genome amplification of the PBMC DNA and sequencing. The sequences were subtyped and each section of the recombinant sequences was annotated with breakpoints using jpHMM^32^.

**Figure 2:**
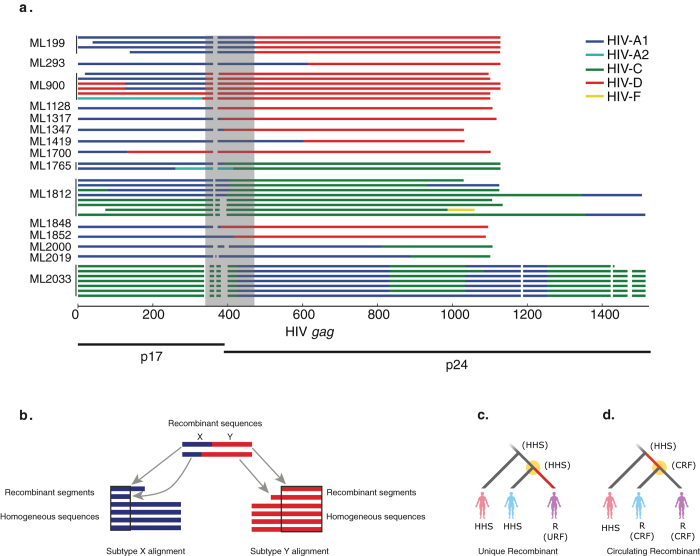
Outline of all unique recombinant HIV-1 gag forms. **a**. Ten women carried one predominant URF, whereas five women carried several distinct URFs (ML199, ML900, ML1765, ML1812, ML2033); in three cases these URFs incorporated sections from additional HIV-1 subtypes (ML900, ML1765, ML1812). Blue = HIV subtype A1 (HIV-A); cyan = HIV subtype A2 (HIV-A2); green = HIV subtype C (HIV-C); red = HIV subtype D (HIV-D); yellow = HIV subtype F (HIV-F); and white = gap. A unique breakpoint pattern was found in patients ML1700 and ML2033. **b.** Outline of the construction of sequence alignments. Subtype-specific alignments (labelled X and Y) were created by dividing the recombinant sequences into subtype-specific sections and using the longest shared subtype-section and matching sections of the HHS sequences from the rest of the women in the cohort and sequences from the HIV database. **c,d**. Outline of the subtype-specific Bayesian phylogenetic analysis based on the assumption that a URF will not recombine again to become an HHS. Any recombinant patient sequence on the tree whose parent node (yellow circle) has an HHS descendant likely is derived from an HHS, which means that the recombinant sequence is likely to be a URF (**c**). If the parent node only has recombinant descendants, then the patient is likely to be infected with a CRF (**d**). The branch on which the recombination event is suspected to have occurred is shown in red.

The following HIV-1 subtypes were prevalent in our cohort: HIV-A1, 61% (69/113); HIV-C, 6% (7/113); and HIV-D, 18% (20/113) (Table S1). These results are consistent with other studies in Kenya^27^,^28^. In addition, 17 women carried recombinant HIV-1 (15%). These recombinant viruses were mixtures of HIV-A1 and HIV-C (n = 5) or of HIV-A1 and HIV-D (n = 12); three patients carried additional recombinant forms (HIV-A2/HIV-D (ML900), HIV-A1/HIV-A2/HIV-C (ML1765), and HIV-C/HIV-F (ML1812)), and two carried recombinants with unique breakpoint patterns (ML1700 and ML2033) (Fig. 2a).

To identify women in whom selection had favoured the outgrowth of URFs, we developed an algorithm to distinguish between URFs and any CRFs that had infected the women by chance. This analysis was based on an examination of subtype-section specific phylogenetic trees constructed from alignments of sequences from this cohort and from the HIV database (as illustrated in Fig. 2b–d and described in the Methods). Briefly, we examined each tree sampled from the posterior distribution for every patient with recombinant HIV-1. For our assessment of whether a given subtype-section was derived from an HHS or a CRF, we assumed that a URF would not recombine again to become an HHS, in contrast to those CTL-escape mutations that confer reduced viral fitness and that typically revert following transmission to HLA-mismatched recipients (reverting mutations)^2^,^33^. Therefore, if a patient’s subtype-section descended directly from a phylogenetic tree node that also had HHS descendants, then we estimated that the patient was infected with a URF (Fig. 2c) because the parent node of an HHS sequence must also have been an HHS. Conversely, if a subtype-section’s parent node had only other recombinant sequences amongst its descendants, then we estimated that the patient was infected with a CRF (Fig. 2d). By performing this analysis on each tree sampled from the posterior distribution of trees, and bringing together the analyses for both segments, we were able to calculate the Bayesian posterior probability that a patient was infected by a URF.

Using the results of the phylogenetic tree and recombinant breakpoint analyses, we estimated that the patient most likely carried a URF if 1) both recombinant sequence-sections were derived from HHSs; 2) one section was derived from an HHS and the other section was derived from a CRF; or 3) the recombinant sequence had a unique breakpoint pattern. Based on this analysis, 15/17 women (88%) were likely to carry a URF and 2/17 carried the same CRF (12%) (Table 1). This CRF was similar to a previously reported recombinant HIV-1 in the HIV database that was derived from Pumwani (GQ431530); thus, this CRF may have been generated and spread locally.

Next, we examined the composition of each URF and found that 6/15 (40%) of the URFs contained sections of two HHSs and that 9/15 (60%) contained one section of a local CRF and one section of an HHS (Table 1). The latter pattern is consistent with a report of a recombinant HIV with sections of a CRF^34^.

This overrepresentation of CRF sections in the URF group is highly unlikely to occur by chance given the frequency of CRFs in the cohort (2/98 (2%) versus 9/15 (60%), Fisher’s exact test, p < 0.0001; odds ratio = 9.8), Thus, our results indicate that local CRFs may have a specific survival advantage in the URF group.

### Overrepresentation of beneficial HLA-variants in HIV-infected women with URFs

To test our hypothesis that unique HIV-1 recombinants are more likely to occur in hosts with CTL responses that are more efficient than average, we compared the frequency of HLA variants associated with delayed disease progression (HLA-A*7401, HLA-B*1302, HLA-B*14/-Cw8, HLA-B*5701, HLA-B*5702, HLA-B*5703, HLA-B*5801 and HLA-B*8101)^2^,^35^,^36^,^37^ in the group of women with URFs to that in women with HHSs/CRFs. These beneficial HLA variants were used as surrogate markers for CTL-responses that were more efficient than average because direct analysis of the women’s CTL responses was impossible due to a lack of PBMC samples. However, a causal relationship between CTL responses and HIV recombination has been demonstrated in one patient^19^ and HLA variants have been successfully used as surrogate markers for CTL-responses in numerous studies, e.g.,^38^.

Women with beneficial HLA variants (12/52) were over 5 times more likely to carry URFs than those without beneficial HLA variants (3/61) (Fisher’s exact test, p < 0.0055; odds ratio 5.7). However, Fisher’s exact test is a test of equality of proportion and, with the proportions in question being based upon URF classifications derived from Bayes Rule, no account has been taken of the uncertainty in those classifications. Although strict classification followed by Fisher’s exact test has the virtues of simplicity and ease of interpretation, information has been discarded; therefore the analyses were improved by fitting binary generalised linear models (GLMs) to the log(odds) of the Bayesian posterior probabilities. Odds ratios quoted for these results refer to a change in Bayesian log(odds) of unity, which corresponds to a posterior probability of 0.75; odds-ratios corresponding to a posterior probability of 0.9 can be approximated by squaring the quoted odds ratio, and cubing it would correspond to a posterior probability of 0.95. This more meticulous approach confirmed that women with beneficial HLA variants were more likely to carry URFs than those without (binary GLM, odds ratio = 1.25, p = 0.0107). Likewise we observed a significantly higher frequency of beneficial HLA variants in the URF group (12/15, 80%) compared with the group with HHSs/CRFs (40/98, 41%) (Fisher’s exact test, odds ratio = 5.7, p = 0.0055; binary GLM, odds ratio = 1.25, p = 0.0107).

These results suggest that the efficient CTL responses associated with beneficial HLA variants selects for the outgrowth of HIV-1 recombinants.

### Beneficial HLA-variants have less beneficial effects on disease progression in HIV-infected women with URFs

**Figure 3:**
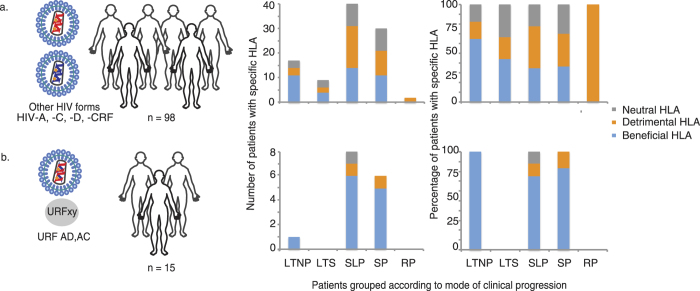
HLA variant distribution in clinical progression groups in women with either HHSs/CRFs or URFs. **a.** The number and percentage of HIV-infected women carrying HHSs or CRFs in clinical groups with distinct disease progression profiles: long-term non-progressors (LTNPs), long-term survivors (LTSs), slow progressors (SLPs), standard progressors (SP) and rapid progressors (RPs). In each group, we indicated the distribution of HLA variants associated with delayed disease progression (beneficial HLA-variants), standard progression (neutral HLA-variants) or rapid progression (detrimental HLA-variants). **b.** As in A, except using data from the group of women carrying unique recombinant forms of HIV-1.

Next, we investigated whether the relationship between beneficial HLA variants and disease progression in the group of women with URFs was similar to that in the group of women with either HHSs or CRFs. We used the biannual CD4-counts to classify the patients into groups according to clinical progression rate and examined the distribution of beneficial, detrimental and neutral HLA variants in each group (LTNPs (HIV-infected for >7 years and stable CD4 counts >400/μl), long-term survivors (HIV-infected for >7 years and stable CD4 counts >200/μl and <400/μl), slow progressors (HIV-infected for 5–7 years and unstable CD4 counts ≥200/μl and <400/μl), standard progressors (HIV-infected for 3–5 years and CD4 counts >200/μl) and rapid progressors (HIV-infected for <3 years and CD4 counts <200/μl)) (Fig. 3).

For both the URF and the HHSs/CRF patient groups, we generated two disease progression (DP) groups; one with long-term control of HIV infection (LTNPs and long-term survivors = DP1) and one with slow/standard disease progression (slow progressors and standard progressors = DP2); rapid progressors (n = 2) were not included because these were found only in the HHS/CRF patient group. We grouped standard progressors and slow progressors because standard progressors often had CD4 counts close to or above 400/μl, consistent with^39^, and the slow progressors counts were unstable.

In the group of women with HHSs/CRFs, we compared the frequency of beneficial, neutral and detrimental HLA variants in DP1 and DP2 by fitting a binary GLM to the HLA variant using neutral as the reference category. We found that beneficial HLA variants predominated in DP1 (binary GLM, odds ratio = 3.24, p = 0.0449), whereas no difference was observed between the frequencies of neutral and detrimental HLA variants (p = 0.3858) (Fig. 3, Table S1). Thus, in women with HHSs/CRFs, those with beneficial HLA variants were over 3 times more likely to have slow disease progression than those with neutral or detrimental HLA variants. If we had included the rapid progressors in DP2, then we would have obtained a slight increase in power (odds ratio = 3.48, p = 0.0328).

Next, we tested whether the presence of either URFs or HHSs/CRFs affected disease progression in patients with beneficial HLA variants, i.e., if the patient would be likely to belong to DP1 or DP2. By regressing on the Bayesian log(odds) we found that patients with URFs were marginally more likely to be in DP2 and that patients with HHSs/CRFs were slightly more likely to be in DP1 (binary GLM, odds-ratio = 1.04, p = 0.0377)(Table S1). Second, we tested if the frequency of beneficial HLA variants was similar in patients with either URF or HHSs/CRFs in DP2 and we observed a significantly higher frequency of beneficial HLA-variants in the URF group (binary GLM, odds-ratio = 1.26, p = 0.0072).

Collectively, these findings suggest that beneficial HLA variants might have less beneficial effects on disease progression in women with URFs than in women with either HHSs or CRFs.

## Discussion

Here, we compared the HLA variant distribution and clinical progression rate in groups of HIV-infected women with or without unique HIV-1 recombinants. Women with URFs had significantly higher frequencies of HLA variants, which were associated with delayed disease progression. In addition, the presence of URFs appeared to decrease the beneficial clinical effect of these HLA variants.

URFs could potentially be generated and selected in most CSWs in our cohort because recombination occurs as a result of the inherent properties of RT, the CSWs likely share the same risk factors, and the risk of HIV superinfection for patients with any given HLA variant is similar to that of initial infection during the first 6 months and subsequently decreases by approximately half^24^,^25^. Thus, the risks of HIV superinfection and inter-subtype recombination are probably greatest during acute and very early infections, consistent with observations in two patients^18^,^19^, but may potentially occur at all disease stages. HIV-1-specific cellular immune responses during chronic infection do not appear to significantly contribute to protection from HIV-1 superinfection^25^ and an overrepresentation of beneficial HLA variants has not been found in patients that were co-infected/superinfected^40^. The only marker that appears to weakly influence the risk of superinfection is the frequency of uninfected CD3+/CD4+/CCR5+ T cells^25^. Effective CTL responses may increase the pool of uninfected CD3+/CD4+/CCR5+ T cells that could be targets for a superinfecting virus, although the findings that CTL activation levels were not associated with the frequency of CD3+/CD4+/CCR5+ T cells^25^ and that beneficial HLA variants were not overrepresented in patients that were co-infected/superinfected^40^ do not support this suggestion.

The significant overrepresentation of patients with beneficial HLA variants in the URF group suggests that the effective CTL responses in these women selects for the outgrowth of the URFs. Because the URFs eventually dominate the viral population, the URFs must be more fit than each of the parent viruses. This fitness gain may result from the selection of HIV-1 with en bloc combinations of advantageous mutations, and recombination may partly explain why HIV-1 containment fails in approximately 70% of patients with beneficial HLA variants within 16 years of infection^41^.

URFs were observed in 23% of women with beneficial HLA variants. This frequency likely represents a near maximum estimate of the frequency of inter-subtype *gag* recombination in this group because the CSWs in our cohort had a very high number of daily customers (an average of 4 men per day) and because the epidemic in Kenya is caused by several co-circulating and relatively prevalent HIV-1 subtypes and CRFs^27^,^28^,^29^,^35^. In combination, these factors provide many opportunities for infection with multiple HIV-1 subtypes, inter-subtype recombination, and CTL-driven HIV-1 adaptation to the host-specific genetic background.

Despite the limitations of our study (i.e., the limited DNA amount obtained from each woman, which constrained the number of analysed Gag copies; the cross-sectional design; the use of HLA variants as surrogate markers for CTL responses and the limited number of women), we still obtained statistically significant results. However, we likely underestimated the overall URF frequency because some patients with homogeneous HIV Gag regions may have viral variants with recombined regions outside Gag. Such recombinants were described by Dowling *et al*., who found that 41% of nearly full-length recombinant HIV-1 sequences from Kenya only contained the recombinant sequence in *pol* and/or env^28^.

Nevertheless, Gag is likely a key target for CTL-driven selection for recombination because only CTL responses against Gag are associated with viral containment and lower mean viral loads^6^. Thus, HIV-1 escape from Gag-specific CTL responses would likely be more advantageous for viral survival and spread than escape from CTL responses that target other viral proteins.

Although CSW infection with a second HIV-1 (HHS or CRF) may be hampered by factors such as anti-HIV immune responses and target cell depletion in the genital tract, it is unclear why most CSWs are not infected with multiple HHSs/CRFs given their daily exposure to numerous clients. One possible explanation is that any superinfecting HIV-1 has to be more fit initially than the established HHS/CRF, which is already adapted to survive in the specific host, and this situation is rarely the case.

The overrepresentation of CRF-sections in HIV recombinants in the URF group suggests that local CRFs may have a specific survival advantage in women with beneficial HLA variants, possibly because the CRFs arise from URFs generated in this patient group. Alternatively, or additionally, a prior successful recombination could be an indicator of subsequent recombination success possibly because mutations in the CRF already compensate for inter-subtype sequence incompatibilities. The potential local CRF fitness advantage and/or inherent CRF recombination propensity further amplifies HIV diversification in individuals with beneficial HLA variants.

Beneficial HLA variants were frequently found in LTNPs and long-term survivors in the HHS/CRF group, whereas beneficial HLA variants were concentrated in slow progressors and standard progressors in the URF group. Thus, the normally protective HLA variants appear to have less of a beneficial effect on disease progression in women carrying URFs. The fact that we predominately observed URFs in women with beneficial HLA variants who had been infected for a short duration and who were progressing faster refutes a key potential source of confounding, namely, that URFs would be overrepresented in HIV-infected women with beneficial HLA variants simply because they are more likely to be superinfected because they survive longer than those without beneficial HLA-variants. Our results are supported by previous reports demonstrating that superinfection and/or dual infection with two HIV strains lead to faster CD4+ T cell decline^42^ and/or to greater increases in viral load^40^ in men who have sex with men, faster disease progression in a mixed cohort^43^ and a trend towards accelerated CD4+ T cell decline and increased viral load in women, although the effect on clinical progression events in the latter study was limited^44^. However, only one of these studies examined whether recombination occurred in the patients’ viral population^40^ and was not powered to estimate what effect recombination had on disease progression. In combination, these studies and our results suggest that the effect of recombination on disease progression needs to be further examined in larger cohorts in which both CD4 counts and viral load data are available and transmitted recombinants could be cloned and tested for fitness *in vitro*.

These results suggest that a balance exists between CTL responses and viral adaptation in most patients with protective HLA variants and that the delayed disease progression in these patients is a result of this unique virus-host equilibrium. However, this beneficial balance may be disrupted if the CTL response results in the outgrowth of highly adapted URFs, and our results suggest that this process limits the protective effects of the immune response. Recombination and outgrowth of unique recombinants could likewise undermine successful intra-host immune containment of other human and animal retroviruses e.g., human T-lymphotropic virus type 1 (HTLV-1)^45^ and Mink cell focus-forming viruses in mice^46^.

Our results suggest that individuals with protective HLA variants who engage in any high-risk behaviour (e.g., prostitution or intravenous drug use) constitute a hub for HIV-1 diversification that could lead to the outgrowth of unique forms of recombinant HIV-1 that have the potential to spread in the population. If these new HIV-1 recombinants do spread, then they will contribute to viral diversification locally and globally and may lead to faster progression to AIDS and/or to more rapid CD4 decline than the homogenous subtypes from which they originated, as observed in, e.g., Guinea-Bissau and Brazil^47^,^48^. Thus, whereas CTL responses in individuals with protective HLA-variants are beneficial for the individual, they may be detrimental to the population because they fuel HIV-1 evolution.

These results suggest that monitoring the effect of HIV-vaccination on HIV-1 diversification in infected vaccinees may be necessary because a vaccine that elicits effective CTL responses, but that does not prevent infection, may increase the formation of URFs and thereby increase viral diversification and adaptation to the vaccinated population.

## Methods

### Study design and participants

We obtained DNA samples extracted from peripheral blood mononuclear cells (PBMC) from 113 HIV-1 infected female CSWs from the Pumwani Sex Worker Cohort^35^. Enrolment in the cohort included a clinical examination, questionnaire and interview completion, HIV-1 testing, lymphocyte counts and written and oral informed consent. The women are followed biannually with blood draws for research studies and lymphocyte counts. All the women in this study were enrolled between 1985 and 1995 and the PBMC samples were obtained between 1997 and 2004, before antiretroviral drugs were available to the cohort. These women constituted all the women in the Pumwani Sex Worker Cohort who fulfilled the following selection criteria: HIV-1 infection, known HIV-1 disease progression profile, no antiretroviral treatment, available DNA sample and known HLA type. The investigators who performed the recombination analyses and allocated the women into groups with different HIV-1 forms (HHS, CRF or URF) were blinded to the HLA data and disease progression profiles. The comparison between HIV-1 form, HLA profile and clinical progression rate was performed by investigators who had not allocated the women into the different HIV-1 form groups. This study was conducted according to the principles expressed in the Declaration of Helsinki; the National AIDS Committee, the National Ethics and Scientific Review Committee of Kenyatta National Hospital and the University of Manitoba Use of Human Subjects in Research review committee have approved studies using this cohort.

### DNA extraction and HLA typing

PBMC DNA was extracted using QIAamp DNA Mini Kits (Qiagen, The Netherlands) and 5–10 μl of each sample was available for this study. HLA class I genotyping was performed using multiplex PCR (Dynal Biotech, Norway).

### Assignment of HLA variants as “beneficial”, “detrimental” and “neutral”

HLA variants were assigned as beneficial, detrimental, and neutral with regard to disease progression based on previously published reports^2^,^35^,^36^,^37^. Beneficial HLA variants included HLA-A*7401, HLA-B*1302, HLA-B*14/-Cw8, HLA-B*5701, HLA-B*5702, HLA-B*5703, HLA-B*5801 and HLA-B*8101. Detrimental HLA variants included HLA-B*07, HLA-B*1510, HLA-B*1801, HLA-B*35, HLA-B*53 and HLA-B5802. All other HLA variants were labelled as neutral. One patient (ML1317) carried two beneficial (HLA-A*7401 and HLA-B*5801) and one detrimental (HLA-B*35) HLA variant and was classified as having an overall beneficial HLA profile (Table 1) because we estimated that the beneficial effect was strongest based on the HLA-distribution results in the group with HHS/CRF, the co-operative additive effect of beneficial HLA variants^49^,^50^,^51^ and previous reports^2^,^35^,^36^,^37^,^38^,^52^. Two patients carried one beneficial and one detrimental HLA-variant (ML293: HLA-B*8101/HLA-B*5802, ML2000: HLA-B*7401/HLA-B*5802) and both carried HLA-C*0602. Because HLA-B*5802 was not associated with detrimental effects in this cohort and because HLA-C*0602 was significantly associated with a decreased risk of seroconversion, although not with slower disease progression^35^, we estimated that the overall HLA profiles were beneficial (Table 1).

### Amplification and sequencing of HIV-1 p24 gag

We amplified ~1200 bases spanning the HIV-1 p17–p24 region using a hot-start nested polymerase chain reaction (PCR) (Advantage 2 PCR Kit; Clontech). The primers were GAG1 (round 1 (R1)) 728–751, GAG3 (R1) 1941–1916, GAG2 (R2) 763-788 and GAG4 (R2) 1911–1884 (nucleotide numbers correspond to HIV_HXB2_ numbering) and annealed to regions conserved amongst the subtypes. Because of limited sample availability, we initially performed bulk DNA amplification to optimize the primers and to identify the patients with mixed sequence reads. Subsequently, we used the PBMC DNA samples to performed single-genome PCR amplification of 5–10 genomes per patient, subject to DNA availability. Forward and reverse sequence reads were assembled and proofread using Pregap4 and Gap4^53^ and aligned using MacClade (Sinauer Associates) (GenBank accession numbers KR781618 - KR782177).

### HIV-1 subtyping and analyses of recombinant HIV-1

All HIV-1 sequences were subtyped, and each section of the recombinant sequences was annotated with breakpoints and its subtype using jpHMM^32^ (Fig. 2a). The recombinant subtype compositions were also examined using REGA v3.0 (RegaSubtyping/stanford-hiv/typingtool; as recommended in^54^), which gave similar results. Because REGA does not explicitly infer breakpoints, we used the jpHMM results in subsequent analyses. Two patients carried recombinants with unique breakpoints (1700 and 2033) and were classified as URFs. In the case of patient ML1700, the breakpoint was outside the p17 and p24 border and combined a homogeneous subtype A sequence with a CRF_D HIV. Similar CRF_D HIV fragments were found in other recombinants (patients ML1317, ML1852 and ML199); however, in these cases, the recombinant breakpoints were found in the p17-p24 border region. In the case of patient 2033, we observed two distinct recombinant HIV variants composed of HIV subtype A and HIV subtype C sequence segments. These variants had multiple additional breakpoints outside the p17-p24 border. These patterns were unique to this patient, and the presence of two related, but distinct, recombinants forms of HIV subtype A and HIV subtype C suggested that the recombination events had occurred in this patient. In the other patients, the breakpoints between subtypes in the conserved p17-p24 border region were too close to classify them as URFs based on the position of the recombination breakpoint (shaded area in Fig. 2a).

To identify women in whom selection had favoured the outgrowth of URFs, we developed a novel phylogenetic algorithm that did not rely on breakpoint patterns to distinguish between URFs and any CRFs that had infected the women by chance. We only used single recombinants (i.e., sequences with one breakpoint) because we needed the longest alignments possible to ensure adequate power in our phylogenetic analyses. Briefly, we constructed separate alignments for each subtype; these alignments consisted of 1) the subtype sections of the recombinant sequences, 2) matching sections of the HHS sequences from our cohort, and, 3) matching sections of the most similar sequences in the HIV database (www.hiv.lanl.gov), as determined by BLASTn^55^ using 1) and 2) as query sequences (Fig. 2b, Table S1, Table S2). Because HIV-1 recombinants with HIV-C had two distinct breakpoints, we constructed two alignments for this subtype. The recombinant sequences had to be split, and each subtype-section had to be analysed in the context of sequences from the same subtype, because each subtype-section in a given recombinant will have a separate evolutionary history, which is highly likely to confound the analysis if the recombinant sequence is included as a whole.

We used Bayesian inferential techniques (MrBayes^56^) to build separate posterior distributions of phylogenies from each of the subtype alignments (HIV-A1 and HIV-C: model K80 + G; HIV-D: model GTR + G). We ran four replicates per analysis for 50 M steps each and confirmed convergence in TRACER. Because the sequences were short and relatively conserved, we were unable to construct maximum clade credibility trees from the posterior trees with sufficient support values on the internal nodes to make URF/CRF calls. Instead, we used a novel algorithm to infer the posterior probability that a patient was infected with a URF directly from the trees sampled from the posterior tree distribution.

We assumed that an inter-subtype recombinant HIV would not recombine again to become an HHS. From this assumption follows that the parent node of any HHS sequence must also be an HHS sequence. Therefore, any tree in which a recombinant sequence has an HHS sister sequence supports the hypothesis that the recombinant sequence is a URF because it is derived from an HHS parent node (Fig. 2c), which would occur if a patient were infected with a homogenous subtype that then recombined within the host to form the URF. Conversely, a recombinant sequence that has another patient’s recombinant sequence as its sister sequence is evidence that both those recombinant sequences are CRFs (Fig. 2d).

Thus, for each tree sampled from the posterior tree distribution derived from a subtype-section alignment (consisting of subtype-sections of recombinant sequences, matching sections from homogenous sequences, and matching sequences from the LANL database) we examined whether the tree supported the URF hypothesis or the CRF hypothesis for each patient. For every patient infected with a recombinant, we identified the common ancestor of the patient’s subtype-section sequences in the tree. If the sequences from that patient were monophyletic (i.e., the common ancestor of the patient’s sequences had descendants from that patient exclusively), then we selected the sequences’ common ancestor’s parent node to assess the recombinant form. If the patient’s sequences were paraphyletic (i.e., the common ancestor of the patient’s sequences had sequences from other patients amongst its descendants), then we selected the common ancestor of the patient’s subtype-section sequences. If the selected node had an HHS from another patient amongst its descendants, then we interpreted the tree as evidence that an HHS had infected the patient and that this HHS had subsequently recombined in the patient to form a URF (Fig. 2c). Conversely, if the selected node only had recombinant sequences amongst its descendants, then we interpreted the tree as evidence that the patient had been infected by a CRF (Fig. 2d).

To ensure that rooting effects did not confound the analysis, we rooted each tree sampled from the posterior distribution on a random sequence from a random HHS-infected patient and excluded patients for that tree whose sequences were too close to the root, i.e., when the selected node had more than four different patient/virus combinations as its descendants. For each patient, we calculated the probability that their sequences in this subtype-section alignment were part of a URF (URF-probability) by dividing the number of trees supporting the hypothesis that the patient was infected with a URF (Fig. 2c) by the number of trees that were not excluded due to rooting effects. Thus, we were able to interrogate the data directly and to integrate over the phylogenetic uncertainty that prevented us from building interpretable trees.

We implemented this algorithm in a Python script using the ete2 tree manipulation module^57^, discarding the first 25% of trees as a burn-in. All analyses were performed separately for each subtype-specific alignment, with the results for each patient’s two subtype-sections only brought together immediately before performing the downstream statistical analysis. URFs can be composed of a fragment derived from a CRF virus joined to a fragment derived from an HHS virus, in which case only the subtype-specific analysis containing the HHS-derived fragment would report the appropriate URF probability. Therefore we only used the higher of the two URF probabilities for every patient when combining the results from the two subtype section analyses performed for that patient. Because no previous analyses of this sort have been done, it was not possible to use an informative prior. The results were expressed as the Bayesian posterior probability that the recombinant HIV sequences in a given patient was a URF (URF maximum probability (MP) in Table 1), and Bayes’ rule was used for classification into HHSs or CRFs (Table 1).

### Statistical analyses

Equality of proportion was tested with Fisher’s exact test. Where we incorporated the uncertainty of the Bayesian sequence analysis, binary GLMs were fitted using the posterior Bayesian log(odds) as an explanatory variable; these values were calculated from the highest Bayesian posterior probabilities of the subtype-sections of a given recombinant HIV-1 (the “URF MP”, Table 1). The two recombinant sequences with unique breakpoint patterns were given a URF probability of a 100. Binary GLMs were also used for comparing the frequency of beneficial, neutral and detrimental HLA variants in DP1 and DP2. All statistical analyses were performed using the R statistical package (version 3.0.0); p < 0.05 was considered significant.

## Additional Information

**How to cite this article**: Chang, C.-H. *et al*. HIV-infected sex workers with beneficial HLA– variants are potential hubs for selection of HIV-1 recombinants that may affect disease progression. *Sci. Rep*. **5**, 11253; doi: 10.1038/srep11253 (2015).

## Supplementary Material

Supplementary Information

## Figures and Tables

**Figure 1:**
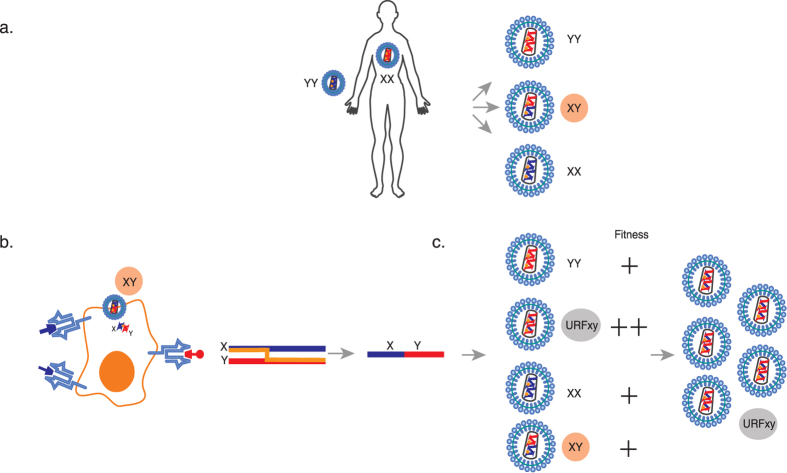
Generation of HIV-1 recombinants and recombinant analyses. **a**. Example of a person infected with two HIV-1 subtypes (subtypes X and Y). Infection with the second HIV subtype (superinfection) may occur at any time after infection by the first HIV subtype but is most likely to occur during the first 6 months of initial infection in women^18^,^19^,^24^,^25^. The two HIV subtypes may infect the same target cell^30^, which can result in the packaging of two different viral RNA genomes into the same virion (highlighted in an orange circle, XY). **b,c**. Examples of infection with the XY virion and of HIV-1 recombination and selection. HIV reverse transcriptase (orange dot in the virion, orange line in the diagram) switches templates between RNA genome X and RNA genome Y to generate a unique recombinant form of the DNA strand (the resulting virion is highlighted in a grey circle, URFxy). Because this recombinant carries the HIV Y escape-mutation version of a targeted CD8 epitope, it is more fit than HIV X, and because this particular sequence combination results in faster replication than HIV Y, it is more fit to replicate and spread in the host than any of the parent viruses. Therefore, URFxy eventually dominates the viral population.

**Table 1:**
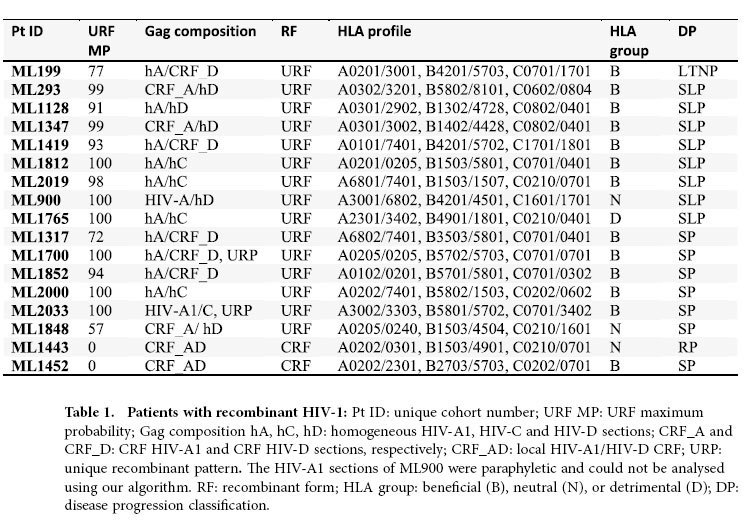
Patients with recombinant HIV-1.
