# Supplementary material for: HIV-infected sex workers with beneficial HLA-variants are potential hubs for selection of HIV-1 recombinants that may affect disease progression
Source: Sci Rep. 2015 Jun 17;5:11253. doi: 10.1038/srep11253 (PMC4469978; doi:10.1038/srep11253)
Supplement: Supplementary Information [file srep11253-s1.doc]

**Supplementary Information**

“HIV-infected sex workers with beneficial HLA-variants are potential hubs for selection of HIV-1 recombinants that may affect disease progression”; Chih-Hao Chang, Nicolaas C. K, Tammy L. Stuart Chester, Vattipally B. Sreenu, Melissa Herman, Ma Luo, Daniel Lunn, John Bell, Frank A. Plummer, T. Blake Ball, Aris Katzourakis, Astrid K.N. Iversen.

1. **Table S1**: HIV subtype, HLA group and disease progression data for patients with HHS or CRFs**.**
2. **Table S2**: List of matching sequence-sections of the most similar *gag* sequences in the HIV Los Alamos database.

**Table S1**

**HIV subtype, HLA group and disease progression data for patients with HHS or CRFs.**

HLA group: beneficial (B), detrimental (D) or neutral (N) HLA variants. DP = disease progression; long-term non-progressor (LTNP), long-term survivor (LTS), slow progressor (SLP), standard progressor (SP) and rapid progressor (RP).

| **ID** | **HIV-1 subtype** | **HLA group** | **DP** |
| --- | --- | --- | --- |
| **5** | C | B | SLP |
| **13** | A1 | D | SP |
| **16** | A1 | D | SLP |
| **24** | C | N | SP |
| **58** | D | B | LTS |
| **59** | A1 | B | SP |
| **115** | A1 | N | SLP |
| **142** | A1 | B | LTS |
| **157** | D | B | LTNP |
| **249** | D | B | LTNP |
| **264** | A1 | D | LTS |
| **265** | A1 | N | LTNP |
| **274** | A1 | B | LTNP |
| **300** | A1 | N | SP |
| **313** | A1 | N | SLP |
| **330** | A1 | N | SP |
| **406** | A1 | B | LTNP |
| **412** | A1 | B | LTNP |
| **415** | A1 | D | LTNP |
| **495** | A1 | N | SLP |
| **525** | A1 | B | LTNP |
| **579** | A1 | N | LTNP |
| **589** | A1 | D | SLP |
| **590** | A1 | D | SLP |
| **602** | A1 | B | SP |
| **646** | A1 | B | LTNP |
| **657** | A1 | N | SLP |
| **672** | A1 | B | LTNP |
| **810** | A1 | N | SLP |
| **874** | A1 | N | SLP |
| **890** | A1 | D | SLP |
| **915** | D | B | LTS |
| **959** | A1 | N | SLP |
| **960** | A1 | N | LTS |
| **977** | D | N | SP |
| **995** | A1 | B | SLP |
| **1032** | A1 | B | SLP |
| **1051** | D | D | SLP |
| **1102** | D | N | SP |
| **1125** | A1 | B | LTNP |
| **1129** | A1 | N | SLP |
| **1197** | A1 | B | SLP |
| **1227** | A1 | B | SLP |
| **1292** | D | B | SLP |
| **1295** | A1 | B | SP |
| **1296** | A1 | D | SP |
| **1318** | A1 | N | SP |
| **1337** | D | N | RP |
| **1346** | A1 | N | SLP |
| **1349** | D | B | SLP |
| **1387** | C | B | SP |
| **1390** | A1 | B | SLP |
| **1401** | A1 | D | LTS |
| **1404** | A1 | D | SLP |
| **1423** | D | B | SLP |
| **1425** | A1 | D | SLP |
| **1443** | CRF_A/D | N | RP |
| **1450** | A1 | N | SLP |
| **1452** | CRF_A/D | B | SP |
| **1474** | A1 | D | SP |
| **1497** | A1 | N | SLP |
| **1514** | D | B | SLP |
| **1535** | A1 | D | SLP |
| **1540** | C | B | SLP |
| **1560** | A1 | N | SP |
| **1561** | D | B | SP |
| **1564** | A1 | B | SLP |
| **1594** | A1 | N | SLP |
| **1596** | C | N | SLP |
| **1611** | A1 | D | SP |
| **1625** | A1 | B | LTNP |
| **1649** | D | B | SLP |
| **1654** | A1 | B | LTNP |
| **1670** | A1 | N | LTS |
| **1725** | A1 | N | LTNP |
| **1731** | A1 | B | SLP |
| **1740** | A1 | D | LTS |
| **1761** | D | N | SLP |
| **1770** | A1 | N | SLP |
| **1771** | D | N | SLP |
| **1772** | A1 | D | SLP |
| **1777** | A1 | B | SP |
| 1802 | C | B | LTS |
| **1811** | D | N | SLP |
| **1825** | C | N | SP |
| **1834** | A1 | N | SP |
| **1859** | D | B | SP |
| **1862** | A1 | D | LTNP |
| **1932** | A1 | B | SP |
| **1947** | A1 | D | SP |
| **1970** | A1 | D | SP |
| **1974** | D | B | SP |
| **2001** | A1 | B | SP |
| **2028** | D | D | SP |
| **2042** | A1 | D | LTNP |
| **2052** | A1 | D | SP |
| **2137** | A1 | D | SP |
| **2204** | A1 | N | SP |

**Table S2**

**List of matching sequence-sections of the most similar *gag* sequences in the HIV Los Alamos database (**[**www.hiv**](http://www.hiv/)**. lanl.gov)**

**HIV-A1:**

A.KE.x.K29.L11770

A1.KE.99.clone_2609.GQ432473

A1.SE.94.SE7253.AF069670

A1D.KE.97.clone_2405.GQ432269_A_frag_1_602

A1.KE.95.clone_2819.GQ432683

A1D.TZ.03.H495.FJ853568_A_frag_1_388

A1.KE.96.clone_1.GQ429865

A1.UG.x.UG031.AB098331

A1.KE.96.clone_2851.GQ432715

A1.KE.98.clone_2547.GQ432411

A1.RW.92.92RW025A.AB287377

AD.KE.98.M381gag.AY772972_A_frag_1_342

A1.TZ.03.H628.FJ853591

A1.KE.00.NKU3005.AF457089

A1.KE.06.06KECst_005.FJ623481

A1.KE.95.clone_1919.GQ431783

01A1.CY.06.CY178.FJ388953

A1D.KE.91.clone_1530.GQ431394_A_frag_1_410

A1D.KE.95.clone_1170.GQ431034_A_frag_1_443

A1.TZ.03.H564.FJ853577

A.KE.99.M439gag.AY772985

A1.KE.95.clone_2101.GQ431965

A1.KE.95.clone_519.GQ430383

A1.KE.95.clone_936.GQ430800

A1D.KE.97.clone_1676.GQ431540_A_frag_1_590

A1.KE.95.clone_2698.GQ432562

A1.UG.03.03_9538NG.AY803390

A1C.KE.96.clone_256.GQ430120_A_frag_1_387

A1.KE.97.ML013_2.AY322185

A.KE.x.K112.L11768

A1.KE.95.clone_2169.GQ432033

A1.KE.92.clone_429.GQ430293

A1.KE.97.ML752.AY322193

A.KE.x.K98.L11775

A1.KE.95.clone_583.GQ430447

A1.KE.95.clone_2518.GQ432382

A1.KE.87.clone_2437.GQ432301

A1.KE.98.clone_2883.GQ432747

A.KE.98.M428gag.AY772986

A1.KE.88.clone_2286.GQ432150

A1.UG.92.92UG037.AB253429

A.KE.98.M369gag.AY772971

A1.KE.01.clone_2639.GQ432503

A1.KE.06.06KECst_007.FJ623476

A1.KE.99.KNH1237.DQ367272

A1.KE.95.clone_41.GQ429905

A1.KE.99.clone_999.GQ430863

A1.KE.02.clone_1129.GQ430993

A.KE.97.M104gag.AY772953

A1.KE.98.clone_1561.GQ431425

A.KE.98.M178gag.AY772958

A1.KE.96.clone_2730.GQ432594

A1.KE.01.clone_220.GQ430084

A1.KE.01.ML1945.EU110088

A1.KE.99.KNH1240.DQ367273

**HIV-C:**

C.CD.x.VI313.L11787

A1C.KE.96.clone_256.GQ430120_C_frag_387_1337

A1C.KE.99.KNH1097.AF457064_C_frag_375_1018

C.TZ.03.H402.FJ853559

C.BR.04.04BR038.AY727524

C.IL.00.00ET669_154_114.AY255817

C.ZM.01.01ZM541036.DQ792998

C.KE.95.clone_1750.GQ431614

C.SO.89.SO145_SM145_SM1451.L11803

C.BW.96.96BW0402.AF110962

**HIV-C:**

C.CD.x.VI313.L11787

C.TZ.03.H402.FJ853559

C.BR.04.04BR038.AY727524

C.IL.00.00ET669_154_114.AY255817

C.ZM.01.01ZM541036.DQ792998

C.SO.89.SO145_SM145_SM1451.L11803

C.BW.96.96BW0402.AF110962

**HIV-D**

D.KE.87.clone_347.GQ430211

D.UG.94.94UG114.U88824

D.KE.88.clone_461.GQ430325

D.KE.89.clone_1093.GQ430957

D.UG.93.93UG_065.AY713418
